# A Rare Case of Trichobezoar Revealing Undiagnosed Celiac Disease

**DOI:** 10.7759/cureus.53919

**Published:** 2024-02-09

**Authors:** Hassnae Tkak, Amal Hamami, Aziza Elouali, Nadir Miry, Amal Bennani, Houssain Benhaddou, Abdeladim Babakhouya, Maria Rkain

**Affiliations:** 1 Department of Pediatrics, Mohammed VI University Hospital, Faculty of Medicine and Pharmacy, Mohamed I University, Oujda, MAR; 2 Department of Histopathology, Mohammed VI University Hospital, Faculty of Medicine and Pharmacy, Mohamed I University, Oujda, MAR; 3 Department of Pediatric Surgery, Mohammed VI University Hospital, Faculty of Medicine and Pharmacy, Mohamed I University, Oujda, MAR

**Keywords:** surgery, trichophagia, adolescent, celiac disease, trichobezoar

## Abstract

Trichobezoar is a relatively rare condition in children, mainly observed in young girls with psychiatric disorders. While documented cases of trichobezoar associated with celiac disease exist, such occurrences remain uncommon in the literature. The association between the two can be explained either by behavioral disorders resulting from a deficiency in iron and folic acid or directly by celiac disease. Treatment is predominantly surgical, and psychological support plays a crucial role in preventing the likelihood of recurrence. We present an unusual case involving the discovery of gastric trichobezoar in a 15-year-old girl who had undiagnosed celiac disease. The condition manifested after she experienced abdominal pain and pallor.

## Introduction

Trichobezoar is an uncommon entity in the pediatric population, occurring in less than 1% of children. It was initially documented in 1779. Trichobezoar is characterized by an atypical accumulation of hair forming solid masses within the digestive tract, most often in the gastric tract [1]. This is explained by the resistance to digestion and peristalsis of human hair due to its smooth surface, leading to its accumulation within the folds of the stomach lining. In 90% of cases, trichobezoar affects young girls suffering from psychiatric disorders, as this condition often arises from a compulsion to pull out their hair (trichotillomania) and ingest it (trichophagia) [1,2]. Its association with celiac disease is well-documented in global literature, yet it is infrequently described. This report details the rare case of a young girl with celiac disease and sheds light on the unusual link between the two conditions.

## Case presentation

A 15-year-old patient had been receiving psychiatric care for trichotillomania with trichophagia for the past four years. Over the last two years, she had experienced pallor and asthenia, which worsened two months ago with the onset of abdominal pain accompanied by postprandial vomiting, without any noticeable changes in bowel movements. All of these symptoms occurred in the context of anorexia, persistent fatigue, and significant, unexplained weight loss.

**Figure 1:**
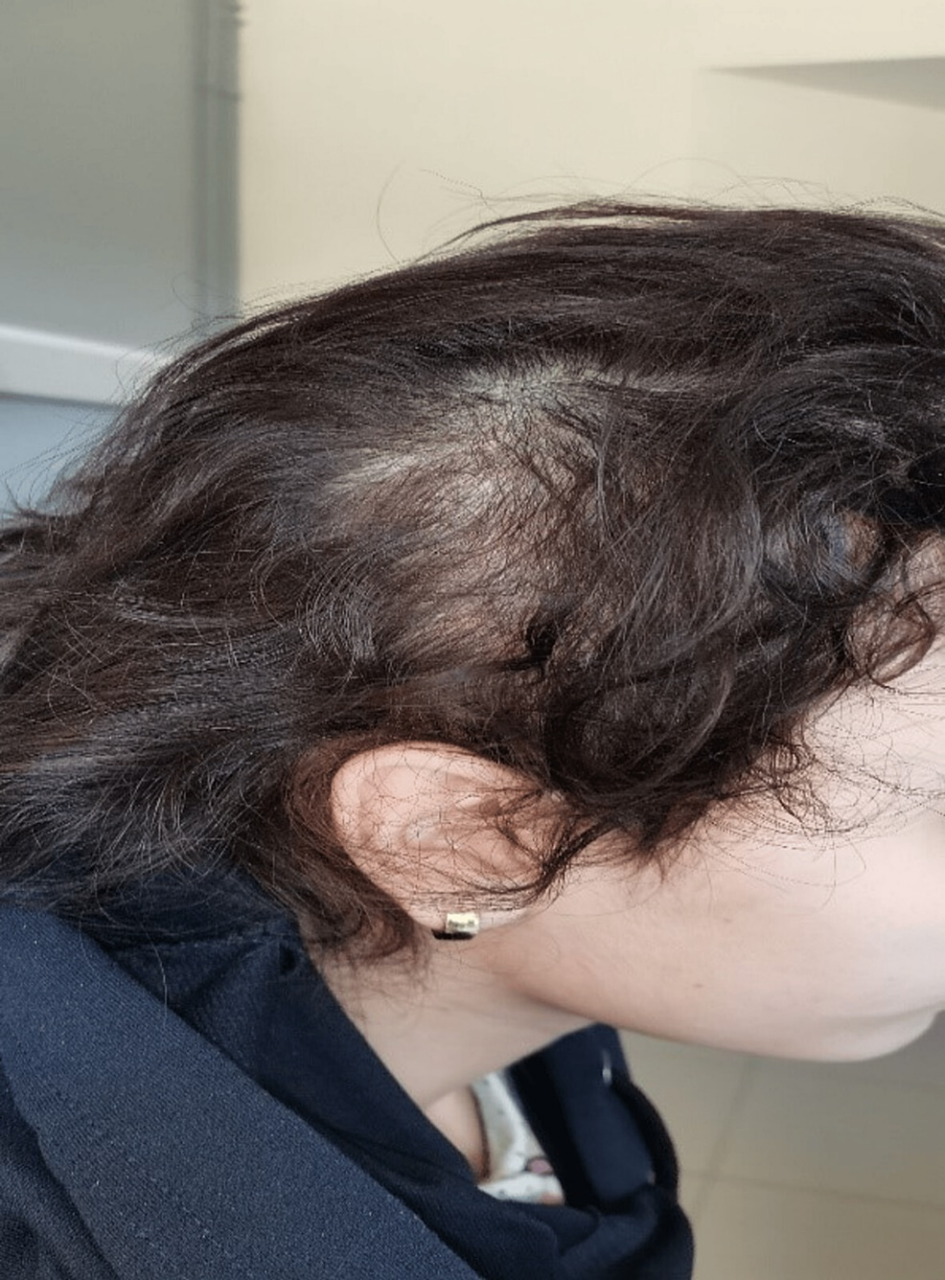
Patches of non-scarring alopecia.

Upon clinical examination, the patient appeared in poor general condition, exhibiting apathy, generalized mucocutaneous pallor, and a growth delay of less than two standard deviations. Abdominal examination revealed a firm, mobile, and painless epigastric mass extending to the right hypochondrium measuring 11 × 6 cm. The scalp exhibited non-scarring hair rarefaction (Figure 1). The rest of the clinical examination did not reveal any notable findings.

**Figure 2:**
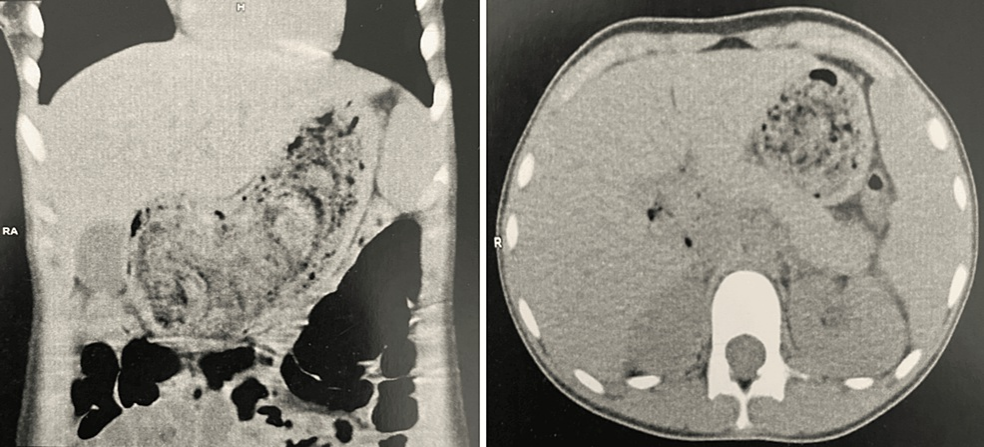
Abdominal CT scan with frontal (left) and axial (right) section showing the presence of an intragastric foreign body, with entrapped air within its tangles, suggesting a trichobezoar.

**Figure 3:**
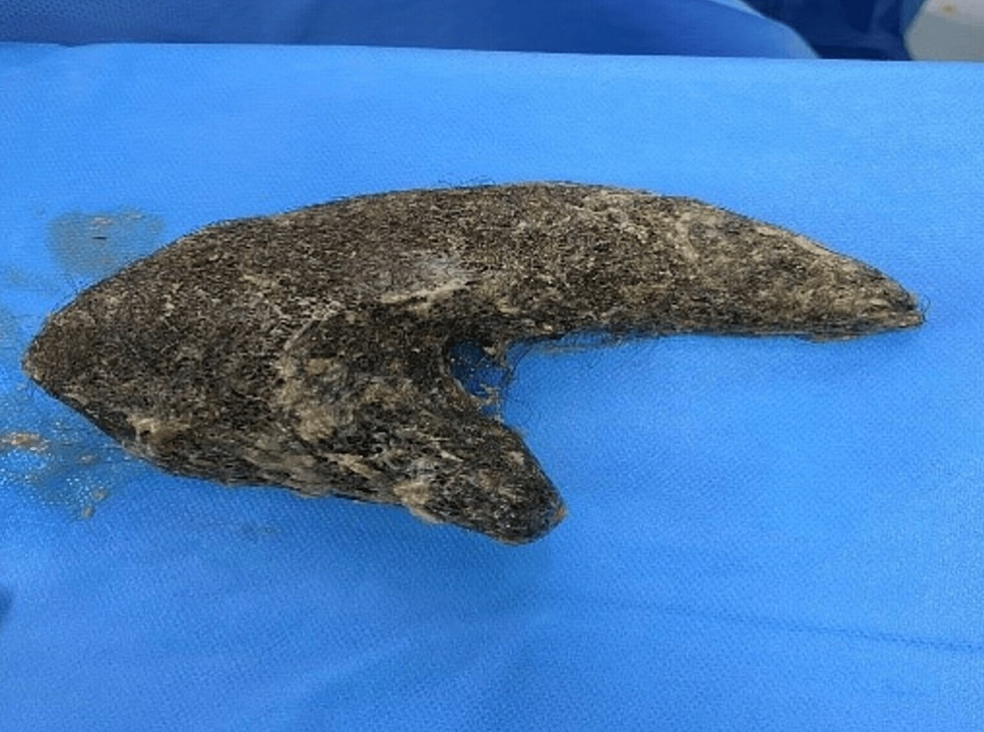
Trichobezoar measuring 9 cm after surgical extraction.

The biological workup revealed microcytic hypochromic anemia, as well as deficiencies in serum ferritin, folic acid, and vitamin D. Abdominal ultrasound showed an attenuating mass measuring 85 mm in the epigastric region. A non-injected abdominal CT scan revealed a distended stomach with a thickened wall containing a large bezoar (Figure 2). The patient underwent median laparotomy for extraction of a giant 9 cm gastric trichobezoar (Figure 3).

**Figure 4:**
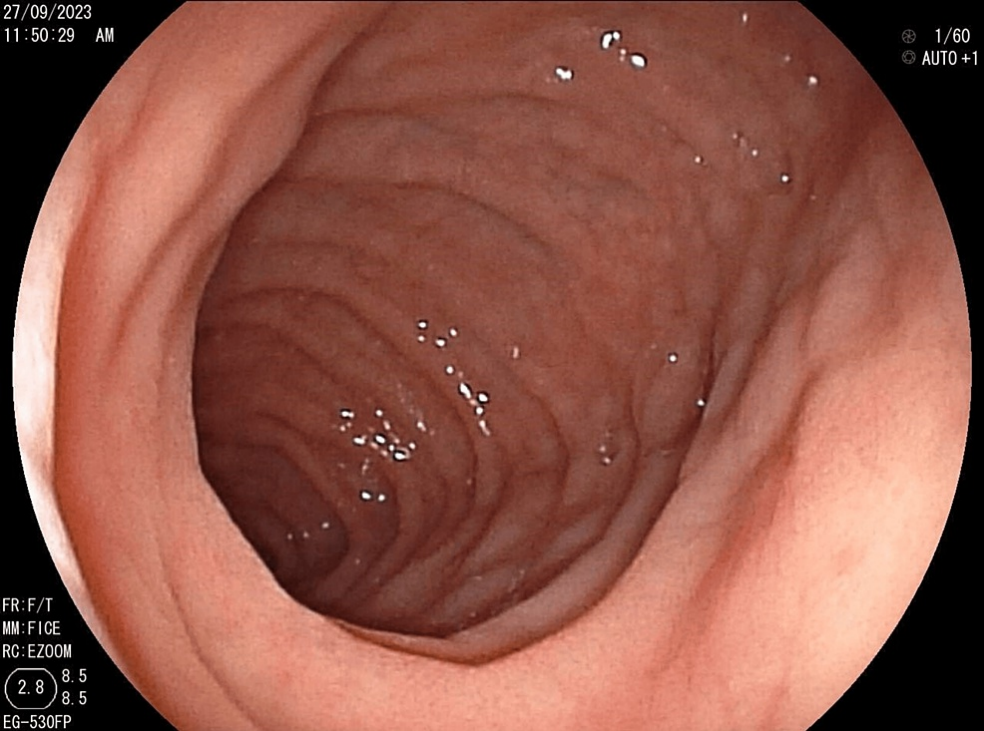
Crenulated appearance of the duodenum.

**Figure 5:**
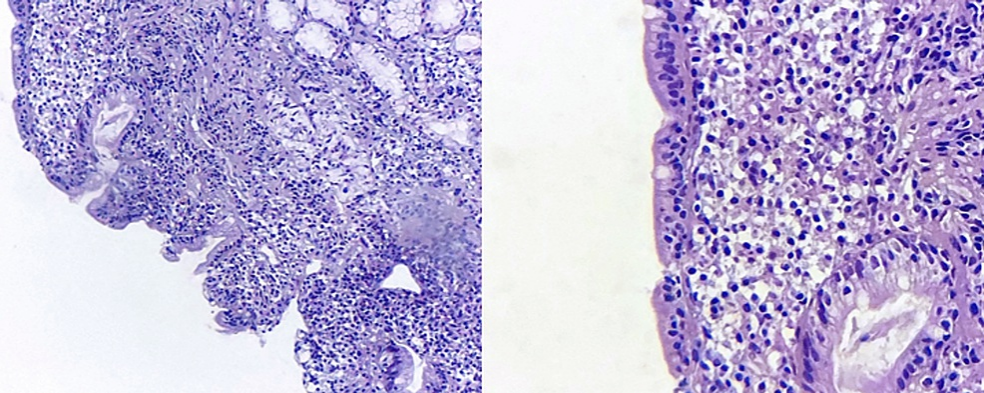
Photomicrographs of the duodenal biopsy show moderately atrophic villi (left: hematoxylin and eosin, ×200), with a considerable amount of intraepithelial lymphocytes (right: hematoxylin and eosin, ×400).

This was supplemented by immunoglobulin A-type anti-transglutaminase antibodies, which were strongly positive, and an esophagogastroduodenoscopy, which revealed a crenelated appearance of the duodenum with antral and duodenal atrophy (Figure 4). Pathology showed moderate villous atrophy with cryptic hyperplasia and intraepithelial lymphocytosis estimated at 34%, compatible with Marsh stage 3a celiac disease (Figure 5). The patient was put on a gluten-free diet with correction of deficiencies and psychological follow-up. The evolution was marked by clinical improvement and the disappearance of behavioral disorders in the days following the initiation of the gluten-free diet.

## Discussion

Bezoar, a rarely described condition in children, is caused by the unusual accumulation of various substances in the digestive tract, particularly in the stomach [1,3]. There are several types of bezoar, depending on the substance ingested. There are lactobezoar, composed of curdled milk and seen exclusively in infants and young children, especially premature babies; phytobezoar, formed by a conglomerate of undigested plant fibers; and trichobezoar, composed of an agglomerate of hair combined with food debris [3-5]. A very rare and specific form of trichobezoar that extends to the small intestine and often leads to serious complications is Rapunzel syndrome, first reported by Vaughan et al in 1968 [1,6]. In children, trichobezoar accounts for 55% of intragastric bezoars. It occurs mainly in adolescent girls aged 10 to 19 years with a history of psychiatric disorders such as trichotillomania and trichophagia [2] as a primary psychiatric disorder, or in association with underlying disorders such as depression, obsessive-compulsive disorder, schizophrenia, autism, pica, and anorexia nervosa [1,3,7].

The association of trichotillomania and trichophagia with celiac disease is well-documented in global literature, yet it is infrequently described. Individuals with celiac disease, whether or not they have anemia, may experience the compulsion to swallow their hair due to deficiencies in iron or folic acid. However, it is noteworthy that trichotillomania and trichophagia might be linked to psychiatric comorbidity arising from celiac disease rather than iron deficiency resulting from the disease itself. Despite documentation [8], only a few cases highlighting this potential association have been reported [3,6-9]. This underscores the significance of our reporting, as it sheds light on the complexity of such manifestations in celiac disease.

Trichobezoar may remain asymptomatic for a long time and discovered incidentally during an etiological workup for iron deficiency anemia or hypoalbuminemia [3]. In its early stages, it may manifest as epigastric discomfort (80%), abdominal pain (70%), nausea or vomiting (65%), asthenia with weight loss (38%), transit disorders (33%), anemia, as well as non-scarring patches of alopecia on the scalp, and eating disorders such as trichophagia [10]. Other revealing features have also been described, such as peptic esophagitis or an abdominal mass. [3,11]. During the course of the disease, sometimes severe complications may arise, such as gastric or duodenal ulceration, upper digestive hemorrhage due to parietal ulceration, mechanical gastric or intestinal obstruction, acute pancreatitis due to obstruction of the ampulla of Vater by a prolongation of the trichobezoar (Rapunzel syndrome), acute intestinal intussusception, digestive fistula, disturbances in absorption, altered general condition with cachexia, digestive perforation, cholestatic jaundice, and, rarely, large intestinal volvulus [3,10,11]. Clinically, in 85% of cases, there is a well-limited abdominal mass in the left hypochondrium and/or epigastrium. The discovery of a patch of localized, non-scarring alopecia is a major sign of trichophagia [10].

The diagnosis is established through esophagogastroduodenoscopy, which remains the recommended examination method, allowing for the visualization of stagnant hairs in the digestive tract. However, in cases of giant forms, it proves insufficient for assessing the extent of the disease, making imaging studies crucial [3,10]. While esophagogastroduodenoscopy may have therapeutic value for extracting small, localized intragastric forms, surgery becomes necessary due to the risk of iatrogenic complications, especially esophageal or intestinal occlusion from trichobezoar fragments [10]. Furthermore, in cases associated with celiac disease, the implementation of a gluten-free diet is essential. Psychological care is also crucial to prevent any recurrence.

## Conclusions

The presence of a firm mass in a pre-pubescent or adolescent girl with trichotillomania suggests the presence of a trichobezoar and consideration should also be given to the possibility of an association with celiac disease. This correlation might be clarified either through behavioral disorders arising from a deficiency of iron and folic acid or directly attributed to celiac disease. Our observation remains distinctive and adds to the infrequent cases documented in the literature. The treatment approach involves surgical extraction of the bezoar, a gluten-free diet in those associated with celiac disease, and a psychological follow-up.
